# Hygiene, Its Agency in Therapeutics

**Published:** 1887-04

**Authors:** B. M. Lawrence


### HYGIENE, ITS AGENCY IN THERAPEUTICS.

BY B. M. LAWRENCE, M. D.

Disease never exists without some cause. Often the most important service the physician can render is to discover, and assist in removing, if possible, the source of ill health, which usually includes some form of impurity. It may exist in the air we breathe, in the food we eat, in the water we drink, and even our thoughts may become so impure that directly they engender vices which result in the most loathsome forms of disease.

"Keep thyself pure," is the best advice possible to give the invalid. The skin, the teeth, the hair, our clothing, bedding and dwellings, should be repeatedly and carefully cleansed.

Whatever obstructs the normal action of any organ, impairs the health. Agencies of this kind have often been at work for a long period of time, and it would require almost a miracle to overcome and remove them all at once. Sometimes, under the best treatment, for days or weeks, but little if any change for the better is apparent, and it frequently occurs that for a while the symptoms appear worse and the patient becomes discouraged, and feels tempted to make a change. This is one of the many reasons why the physician, like the preacher, should be employed by the year. He would then have every inducement to immediately effect a radical cure, and would not be misunderstood in his endeavors to prevent the sufferer from over doing, by taking too much of the treatment, too much food, or too much exercise. In these cases it is better to let the system have a day or two of rest, and cease to urge the life forces. Patience is a virtue which the afflicted must learn to practice in order to recover lost health. A good rule for invalids to adopt, with regard to exercise, either physical or mental, is never to attempt more than half what they feel fully able to perform, and always stop eating before the food has entirely lost all relish. Partake of wholesome, easily digestible food, prepared in the simplest and plainest manner. Eat very slowly and masticate thoroughly. Avoid everything which experience proves to be injurious, or that causes distress in the stomach, trouble with the heart, or is calculated to constipate the bowels, including such articles as fine flour bread, cake, cheese and fried meats.

Wheat is the most perfect article of food ever produced by the bountiful hand of Nature. The whole grain is preferable. It should be boiled or steamed *five or six hours*, until it becomes soft. If boiled tender and then baked or parched it is much superior to that prepared by any other process; and from the fact that it can be masticated it will be found superior to any of the numerous mushes, puddings and porridges so often recommended to children and invalids. The boiled or parched grain may be relished with a little cream and jelly or sugar, or eaten with milk and honey or molasses. The boiled wheat, combined with stewed tomatoes or prunes, apples, peaches, pears or other fruits, will prove both food and medicine, *never failing to cure constipation*, which, if not checked in time, leads to some serious diseases of the kidneys, nerves, or vital organs. Wheat meal should be substituted for the fine flour; it may be combined with oat meal, rye or corn meal, or equal parts of each may be made into bread, biscuit, cakes, crisp crackers or mushes. Graham gems may be made by mixing an egg beaten with milk, to form a batter out of the mush or the unbolted meal, and baked in *very hot cast iron pans.* Heat the pans hot before putting in the batter, and, if the oven is well heated, they will be sufficiently light without adding any baking powder, soda or potash. Stale bread is more healthful than that which is fresh made. It may be steamed or toasted, and if made of coarse flour or meal, is equally good for invalids and those in health. Cream and sauce or a cream gravy is better than butter to be used on cakes, gems or bread.

The people of Boston should not be permitted to monopolize the luxury of baked beans, which, when properly prepared, are very nutritious and palatable. They should be soaked until swelled, then parboiled in fresh water till soft, after which they should be baked from six to twelve hours. If boiled wheat, hominy or corn is added with the beans before they are cooked, a most perfect form of food will be secured. A small piece of fat corned beef or a lump of butter is a superior substitute for the fat pork so generally used.

Vegetables, including carrots, as perhaps the head of the list for making pure blood, when properly prepared, form a most important part of any well regulated bill of fare of a health-promoting dinner:

Fruits should not be taken with vegetables. Animal food should not be indulged in too freely, perhaps the less the better in most cases. "Swine's flesh thou shalt not eat," was the law for the Jews, and should be for the Gentiles. Meats should never be fried. Thin pieces of lean meat may be baked or parched like a griddle cake, turning them over till brown on both sides. For bad cases of dyspepsia, meats prepared in this manner often prove of great benefit.

Fresh fish will be found to agree with most invalids. Eggs, properly prepared, are among the best forms of animal food. Milk is somewhat constipating, and should not be used without adding one-eighth of lime water.

"Temperance in all things," should be the motto of all who wish to retain or regain the boon of health. Sometimes it is well to fast for a meal or for days, especially so when suffering pain from any severe rheumatic or neuralgic trouble. Many sick people have a morbid rage for food, which is not a natural appetite. All such, and in fact most sick people, would improve much faster with only one or two meals a day, taken at regular times, using nothing but water or milk and water between meals. What to drink will be considered in the next article.

